# Intraspecific variation in the karyotype length and genome size of fungus-farming ants (genus *Mycetophylax*), with remarks on procedures for the estimation of genome size in the Formicidae by flow cytometry

**DOI:** 10.1371/journal.pone.0237157

**Published:** 2020-08-06

**Authors:** Mariana Neves Moura, Danon Clemes Cardoso, Brenda Carla Lima Baldez, Maykon Passos Cristiano

**Affiliations:** 1 Programa de Pós-graduação em Ecologia, Universidade Federal de Viçosa, Viçosa, Minas Gerais, Brazil; 2 Departamento de Biodiversidade, Evolução e Meio Ambiente/ICEB, Universidade Federal de Ouro Preto, Ouro Preto, Minas Gerais, Brazil; 3 Programa de Pós-graduação em Ecologia de Biomas Tropicais, Universidade Federal de Ouro Preto, Ouro Preto, Minas Gerais, Brazil; University of North Carolina at Greensboro, UNITED STATES

## Abstract

Ants (Formicidae) present considerable diversity in chromosome numbers, which vary from n = 1 to n = 60, although this variation is not proportional to that in genome size, for which estimates range from 0.18 pg to 0.77 pg. Intraspecific variation in the chromosome number and karyotype structure has been reported among species, although the variation among populations of the same species has received much less attention, and there are few data on genome size. Here, we studied the karyotype length and genome size of different populations of the fungus-farming ants *Mycetophylax conformis* (Mayr, 1884) and *Mycetophylax morschi* (Emery, 1888). We also provide remarks on procedure for the estimation of ant genome size by Flow Cytometry (FCM) analysis. Chromosome number and morphology did not vary among the populations of *M*. *conformis* or the cytotypes of *M*. *morschi*, but karyotype length and genome size were significantly distinct among the populations of these ants. Our results on the variation in karyotype length and genome size among *M*. *morschi* and *M*. *conformis* populations reveal considerable diversity that would be largely overlooked by more traditional descriptions of karyotypes, which were also supported by the estimates of genome size obtained using flow cytometry. Changes in the amount of DNA reflect variation in the fine structure of the chromosomes, which may represent the first steps of karyotype evolution and may occur previously to any changes in the chromosome number.

## Introduction

The eukaryote genome is packed inside the nucleus and comprises from one to many DNA molecules that together correspond to the chromosome set or karyotype of the species. The genome size (GS, also known as the DNA content or C-value) of an organism is the total amount of DNA contained in its haploid chromosome set [1] and is measured in picograms (pg) or the number of base pairs (bp) [2]. The GS is an important trait because it corresponds to the bulk amount of DNA, and is a unit of measurement that is considered to be one of the most fundamental properties of any organism [1, 3].

Ants (Formicidae) present considerable variation in chromosome number, varying from n = 1 in the Australian bulldog ant, *Myrmecia croslandi* [4], to n = 60 in the giant Neotropical ant *Dinoponera lucida* [5]. Even so, genome sizes appear to be much less variable (three times less) ranging from 0.18 pg in *Dorymyrmex bureni* (subfamily Dolichoderinae) and *Paratrechina longicornis* (subfamily Formicinae) [6] to 0.77 pg in *Solenopsis invicta* (subfamily Myrmicinae), the largest estimate obtained up to now [7], although different estimates have been obtained for *S*. *invicta* using alternative methods of evaluation [8].

While chromosome number does not appear to be correlated with genome size (see [9]), interspecific karyotype variation in the Formicidae appears to be related to the age of the lineage [10, 11]. In ancient clades, such as the poneromorph subfamilies, major differences in chromosome number exist between species or even within populations (e.g. [12]). By contrast, the chromosome number appears to be more stable in “recently split” ant lineages, such as the leafcutter ants [13]. Similarly, genome size may correlate with the chromosome number when considering a specific group or taxa [9, 14].

The karyotypes of at least 760 ant species have been analyzed up to now [9, 15], although most studies are limited to a basic description of chromosome number and morphology. So far, only a few studies have investigated the chromosomal variation at the population level (*e*.*g*. [11, 16–18]), and even fewer have applied a karyomorphometrical approach [18–20]. Karyomorphometry is a technique that has been applied amply in cytogenetic studies of plants, and is often combined with estimates of genome size, i.e. [21, 22]. The first studies that measured ant chromosome complements were those of Palomeque et al. [23], Lorite et al. [24], and Lorite et al. [25], although none of these studies evaluated variation at the population level nor they were combined with estimates of genome size. Analyses of this type are available for parasitic wasps, however [26, 27], and these studies have shown that the genome size of the study species was correlated with karyotype length in the families Aphelinidae and Figitidae. Studies of this type provide important insights, from both intra- and interspecific perspectives, on the hidden variation in chromosome structure and karyotypes.

In the present study, we examine the intraspecific and intra-colony variation in genome size and the chromosomal features of two closely-related species of fungus-farming ants of the genus *Mycetophylax*. We used a karyomorphometrical analysis to characterize the karyotypes of geographically distinct populations of *Mycetophylax conformis* (Mayr, 1884) and *Mycetophylax morschi* (Emery, 1888). We complemented the chromosomal analyses with estimates of genome size obtained by flow cytometry (FCM). Here, we document variations in karyotype length that match the differences in genome size estimated for the different studied populations. We also provide methodological remarks for the estimation of ant genome size by FCM, in accordance with the internal standards, the lysis buffers, and the tissues used to obtain the nuclei suspensions.

## Materials and methods

### Colony sampling

Colonies of the fungus-farming ants *Mycetophylax conformis* (Mayr, 1884) and *Mycetophylax morschi* (Emery, 1888) were sampled at different localities and analyzed by karyomorphometry and flow cytometry (FCM). Samples were collected from a total of 36 colonies (Table 1) during field expeditions in the Brazilian states of Bahia (BA), Minas Gerais (MG), Rio de Janeiro (RJ), Santa Catarina (SC), and Rio Grande do Sul (RS). The colonies were excavated according to protocol of [28]. In each case, the whole colony was collected, transported to the laboratory, and maintained under laboratory conditions. During some field expeditions, specimens of other species of ant, including fungus-farming ants and species of other formicide subfamilies, were also collected to evaluate the lysis buffers (see below). These species were *Mycetophylax simplex* (Emery, 1888), *Mycetomoellerius holmgreni* (Wheeler, 1925), *Ectatomma brunneum* Smith, 1858, *Neoponera marginata* (Roger, 1861), *Pseudomyrmex gracilis* (Fabricius, 1804), and *Pseudomyrmex schuppi* (Forel, 1901). The *Mycetomoellerius* and *Mycetophylax* species were identified using the taxonomic keys of Klingenberg and Brandão [29] and Mayhé-Nunes and Brandão [30]. In all cases, vouchers of each species were stored in absolute alcohol and sent to Dr. Rodrigo Feitosa (Universidade Federal do Paraná, Brazil) for identification. Estimates of the size of the genomes of the *Mycetophylax* species were obtained from Cardoso et al. [14].

**Table 1:** Ant colonies sampled during the field expeditions conducted in the present study in eastern and southern Brazil, estimates of the genome size of the species analyzed, and the type of buffer used for the extraction of the nuclei.

Species	Voucher	Locality—State	GS (pg)*	Mean GS (pg) ± SD**	GS (Mbp)	Buffer
*Ectatoma brunneum*	GNSP0167	Cabo Frio—RJ	0.400	0.40 ± 0.010	391.2	LB01
*Mycetophylax conformis*	MYCO0192	Ilhéus—BA	0.276	0.28 ± 0.005	273.8	Galbraith
	MYCO0187	Sargi—BA	0.287			
	MYCO0151	Cabo Frio—RJ	0.326	0.33 ± 0.013	322.7	Galbraith
	MYCO0154	Peró—RJ	0.344			
	MYCO0149	Arraial do Cabo—RJ	0.337			
*Mycetophylax morschi*	MYMO0193	Itacaré—BA	0.363	0.35 ± 0.008	342.3	Galbraith
	MYMO0191	Pontal—BA	0.347			
	MYMO0148	Cabo Frio—RJ	0.374	0.37 ± 0.013	361.9	Galbraith
	MYMO0158	Cabo Frio—RJ	0.355			
	MYMO0247	Araranguá—SC	0.332	0.33 ± 0.001	322.7	Galbraith
	MYMO0188	Araranguá—SC	0.332			
	MYMO0184	Torres—RS	0.325	0.33 ± 0.008	322.7	Galbraith
	MYMO0183	Torres—RS	0.333			
*Mycetophylax simplex*	MYSI0182	Araranguá—SC	0.394	0.40 ± 0.023	391.2	Galbraith
	MYSI0146	Araranguá—SC	0.408			
	MYSI0144	Araranguá—SC	0.392			
*Neoponera marginata*	POSP0201	Cachoeira do Campo—MG	0.649	0.65 ± 0.005	635.7	LB01
*Pseudomyrmex gracilis*	POSP0164	Cachoeira do Campo—MG	0.407	0.41 ± 0.010	401.0	Galbraith
*Pseudomyrmex shuppi*	POSP0202	Cachoeira do Campo—MG	0.376	0.38 ± 0.016	371.6	Galbraith
*Mycetomoellerius holmgreni*	TRHO0197	Cachoeira do Campo—MG	0.335	0.32 ± 0.019	313.0	Galbraith
	TRSP0098	Cachoeira do Campo—MG	0.294			
	TRTH0489	Cachoeira do Campo—MG	0.334			
	TRTH0489	Cachoeira do Campo—MG	0.332			
	THMC0018	Araranguá—SC	0.302	0.31 ± 0.013	303.2	Galbraith
	THMC0066	Araranguá—SC	0.317			
	TRHO0404	Araranguá—SC	0.301			
	THMC0059	Araranguá—SC	0.335			
	THMC0064	Araranguá—SC	0.306			
	THBG0041	Bal. Gaivota—SC	0.380	0.35 ± 0.017	342.3	Galbraith
	THBG0044	Bal. Gaivota—SC	0.335			
	THBG0049	Bal. Gaivota—SC	0.371			
	THBG0054	Bal. Gaivota—SC	0.337			
	THBG0052	Bal. Gaivota—SC	0.324			
	THBG0032	Bal. Gaivota—SC	0.332			
	THBG0031	Bal. Gaivota—SC	0.343			
	THTO0023	Torres—RS	0.333	0.35 ± 0.009	342.3	Galbraith
	THTO0020	Torres—RS	0.348			
	THTO0070	Torres—RS	0.361			
	THTO0071	Torres—RS	0.347			

Samples were collected between October 2015 and September 2017. Brazilian states: RJ = Rio de Janeiro; BA = Bahia; SC = Santa Catarina; RS = Rio Grande do Sul GS: Genome Size; pg: picograms; SD: standard deviation; Mpb: Megabase pairs;

*Mean per specimen of the colony;

**Mean per colony.

### Cytogenetic and karyomorphometric analyses of *Mycetophylax*

Metaphase chromosomes were obtained from *Mycetophylax conformis* collected in Rio de Janeiro, and the *Mycetophylax morschi* cytotypes were defined according to the method proposed by Imai et al. [31]. The analyses were conducted on preparations obtained from 15 individuals per colony. The preparations were stained with Giemsa diluted in Sörensen buffer at (4%) for 20 minutes. On average, 10 metaphases were analyzed per slide. The best metaphases were photographed using an Olympus BX53 microscope equipped with a DP 73 Olympus^®^ camera. The chromosome morphology was evaluated using the karyomorphometrical approach described by Cristiano et al. [20]. For this, we used the Image Pro Plus^®^ software (Media Cybernetics, Rockville, MD) to measure each individual chromosome from the centromere to the end of the long (L) and short arms (S), as well as the total length (TL) of the chromosome. Chromosome length was averaged for the 10 individuals measured from each colony. The sum of the lengths of all the individual chromosomes constitutes the karyotype length (KL). The chromosomes were classified as metacentric (M), submetacentric (SM) or acrocentric (A) based on the arm ratio of Levan et al. [32] with the nomenclatural adjustments of Crozier [33].

## Genome size analyses

### Evaluation of the internal standards and lysis buffers

Two internal standards are commonly used to estimate genome size in hymenopteran species, in particular in ants and stingless bees. These standards are *Drosophila melanogaster* and *Scaptotrigona xantotricha* (e.g. [6, 14, 34–36]). *Scaptotrigona xantotricha* was initially described as the internal standard by Lopes et al. [37]–the mean genome size of the females is 1C = 0.44 pg = 430 Mb. The *Drosophila melanogaster* wild type (Oregon-R) reared in our laboratory at the Federal University of Ouro Preto was first tested against *S*. *xantotricha*. This analysis confirmed that the mean size of the genome of our *D*. *melanogaster* strain is 1C = 0.18 pg = 175 Mb (see S1 Fig) [37]. Both these standards were tested so that we could determine the internal reference standard for estimating the nuclear genome size of the ants collected in the present study.

We also tested the three most widely used nuclear isolation buffers, two of which (OTTO I/OTTO II and Galbraith) are most used for the order Hymenoptera. The other buffer, LB01, is commonly used in plant studies (Table 2) [38, 39]. The selection of the buffers was based on the findings of Loureiro et al. [39], who determined that these three buffers, due to their different chemical compositions, produced different results, depending on the species being analyzed. We used the three buffers in our analyses as described originally and optimized by the respective authors (see Table 2).

**Table 2:** Three nuclear isolation buffers tested in the present study.

Buffer	Composition	References
Galbraith	45 mM MgCl_2_, 30 mM sodium citrate, 20 mM MOPS, 0.1% (v/v) Triton X-100, pH 7.0	Galbraith et al. [40]
LB01	15 mM Tris, 2 mM Na_2_EDTA, 0.5 mM spermine.4HCl, 80 mM KCl, 20 mM NaCl, 0.1% (v/v) Triton X-100, 15 mM *β*-mercaptoethanol, pH 7.5	Doležel et al. [41]
OTTO’s	OTTO I: 100 mM citric acid monohydrate, 0.5% (v/v) Tween 20 (pH 2–3)	Otto [42]
	OTTO II: 400 mM Na_2_PO_4_.12H_2_O (pH 8–9)	Doležel & Göhde [43]

MOPS = 4-morpholinepropane sulfonate; DTT = dithiothreitol; Tris = tris-(hydroxymethyl)-aminomethane; EDTA = ethylenediaminetetraacetic acid. (modified from Loureiro et al. [39]).

### Preparation of the tissue and samples, and the flow cytometry (FCM) analyses

We used the heads of adult workers of the study species to determine the amount of DNA. Both internal standards and the three buffers described above were tested in each ant species. The heads were severed with a scalpel blade, placed in a 1.5 mL microtube, and immersed in 100 μL of the buffer. Some of the experiments were run using ganglia from pupae with the white compound eyes typical of the study species, in order to verify if there were differences in the genome size estimates obtained from the ganglion tissue between adults and pupae. The samples and internal standard were ground with a pestle, with the microtube was shaken vigorously up and down to detach the cells from the tissue and release the nuclei. The buffer (600 μL) was then added to the microtubes. In the specific case of the OTTO buffer, an additional centrifugation step was applied, followed by cell resuspension [44]. The nuclei suspension of each sample was filtered through a 40 μm nylon mesh and stained with 6.5μL of propidium iodide (PI) solution at a concentration of 1.0 mg/mL. Exactly 3.5 μL of RNAse (10mg/mL) was also added to each sample, and the samples were then stored in the dark at 4°C for 30 minutes prior to analysis. In the trials of the Galbraith and LB01 buffers, the samples were stained for 10 min, whereas in the OTTO trials, they were stained immediately.

The analysis was run in a FACSCalibur (Becton Dickinson) cytometer at the Federal University of Ouro Preto. This cytometer was equipped with a laser source (488 nm), and the histograms were obtained using the Cell Quest software. A minimum of 10,000 nuclei were counted from each sample, and the relative intensity of their fluorescence was analyzed with the cytometer configured to run at low speed. Three independent replicates (three specimens per colony) were analyzed. The histograms with a coefficient of variation (CV) of over 5% were rejected and a new trial was run. The histograms were visualized in the Flowing 2.5.1 software (http://www.flowingsoftware.com). The size of the genome of each sample was calculated using the 1C values for either *D*. *melanogaster* (0.18 pg) or the *S*. *xantotricha* female (0.44 pg), and the estimates were obtained according to the equation of Doležel and Bartoš [38]. The final value was calculated based on three replicates per species per colony. These values were then converted to megabase pairs (1 pg = 978 Mbp) [2].

## Statistical analyses

General linear models were constructed to verify the differences between the mean genome sizes of the sample colonies of *Mycetophylax morschi* and *Mycetophylax conformis*. The variation in the mean genome size among the species and colonies was assessed by an Analysis of Variance (ANOVA) of the General Linear Model, followed by a contrast analysis with an *alpha* of 5%, when the *p* value of the GLM-ANOVA was significant (p < 0.05). The differences between the mean genome sizes obtained using the different buffers were also evaluated, as well as those between the pupae and the adult workers.

To evaluate the efficacy and reliability of the flow cytometry, and validate the genome size estimates, the coefficient of variation (CV) was calculated between the days of measurement by using samples from the same colonies as the quality control. Loureiro et al. [39] found variation between different days of measurement and also between analysts, but as the same analyst examined all the samples in the present study, only the effect of the day was evaluated here. This “statistical” CV is different from that generated in the analysis of the histograms, being calculated by dividing the standard deviation (SD) by the mean genome size of the species analyzed on each day of measurement. The statistical CV evaluates the degree of variation among trials. All the colony estimates of the four myrmicine species were included in this analysis. The differences between the mean statistical CV for each day were assessed by an Analysis of Variance (ANOVA) of the general linear models (GLMs) followed by a contrast analysis with an *alpha* of 5%. All the statistical analyses were run in R v2.15.1 [45] and a residual analysis of the GLM was undertaken so that the error distribution could be evaluated [46].

## Results

### Karyotype and genome size

**Fig 1:**
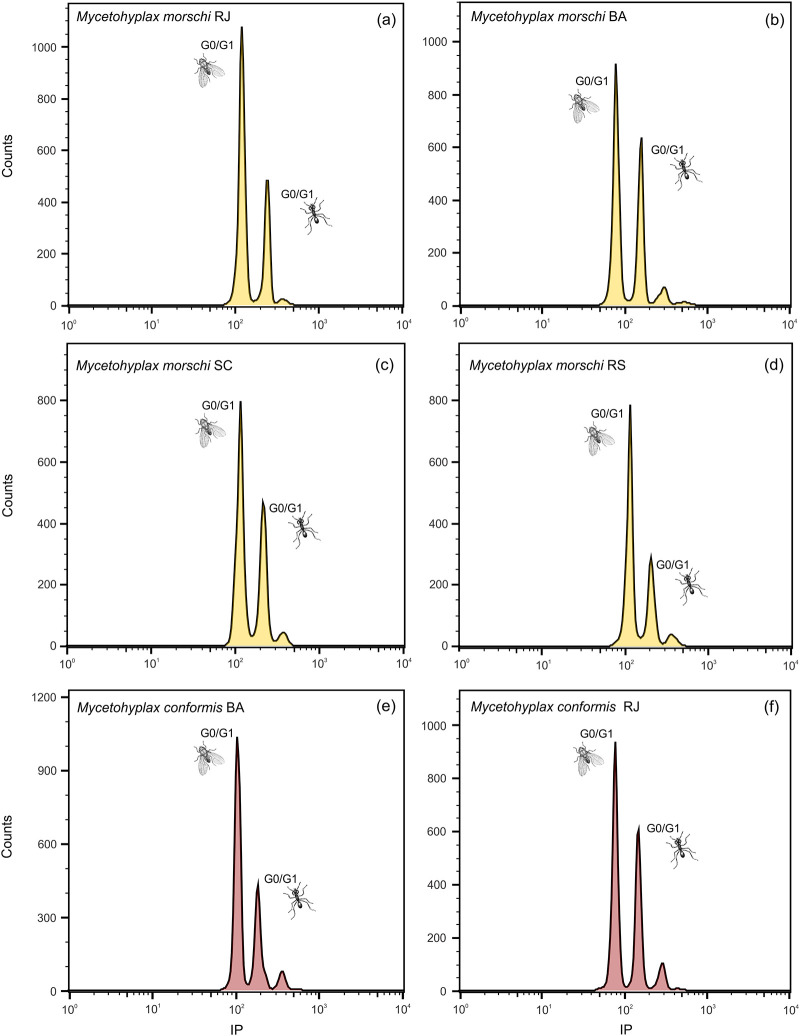
Histograms of the genome sizes of the *Mycetophylax* species based on the analysis of the nuclear suspensions of the adult head tissue stained with propidium iodide (PI). (a) *M*. *morschi* from the state of Rio de Janeiro—RJ (2C = 0.64 pg) and *Drosophila melanogaster* (internal standard 2C = 0.36 pg). (b) *M*. *morschi* from the state of Bahia—BA (2C = 0.72 pg) and *D*. *melanogaster* (internal standard 2C = 0.36 pg). (c) *M*. *morschi* from the state of Santa Catarina—SC (2C = 0.64 pg) and *D*. *melanogaster* (internal standard 2C = 0.36 pg). (d) *M*. *morschi* from the state of Rio Grande do Sul—RS (2C = 0.64 pg) and *D*. *melanogaster* (internal standard 2C = 0.36 pg). (e) *M*. *conformis* from the state of Bahia—BA (2C = 0.56 pg) and *D*. *melanogaster* (internal standard 2C = 0.36 pg). (f) *M*. *conformis* from the state of Rio de Janeiro—RJ (2C = 0.64 pg) and *D*. *melanogaster* (internal standard 2C = 0.36 pg).

**Fig 2:**
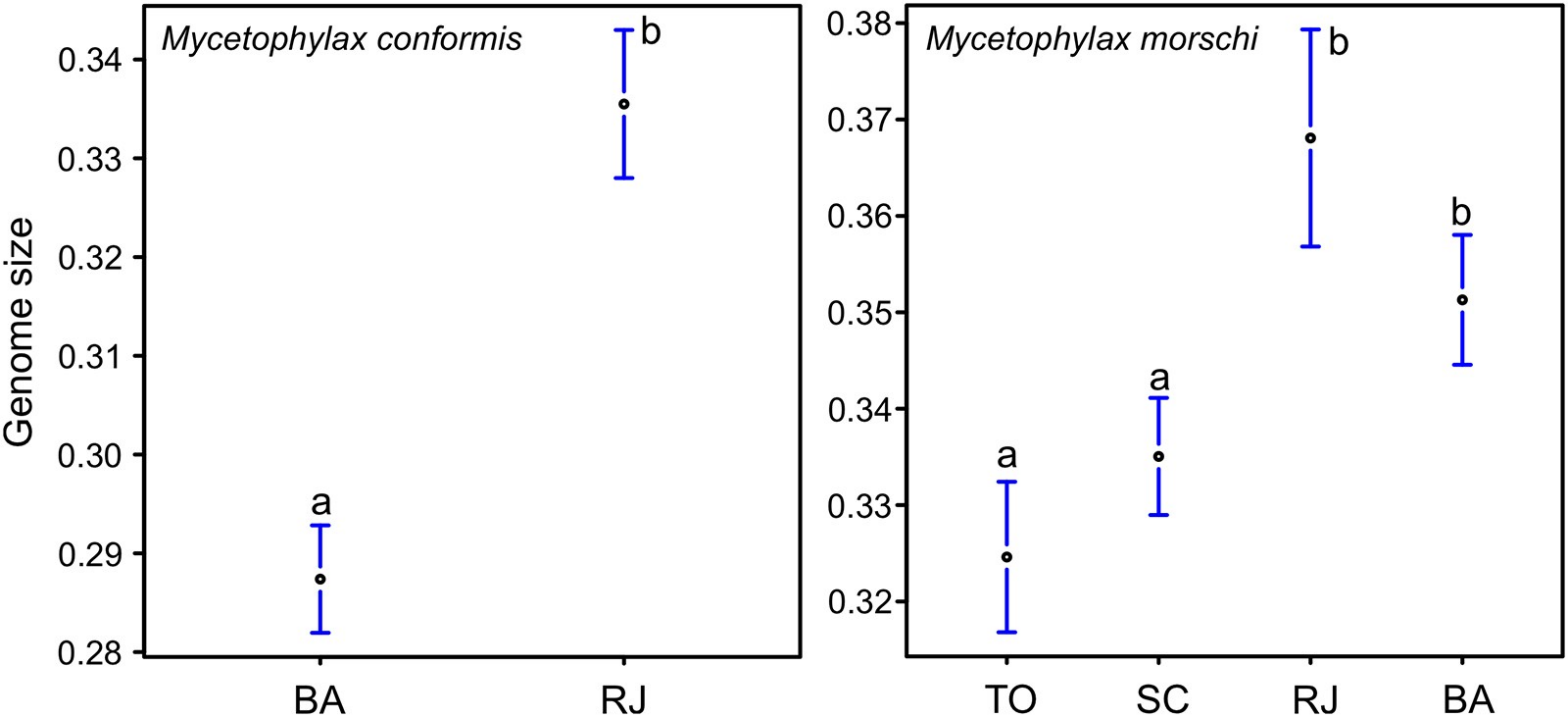
The range of genome sizes (GS) estimated for (a) *Mycetophylax conformis*, and (b) *Mycetophylax morschi*. The dots represent the mean, and the bars, the confidence intervals. The *x* axis corresponds to the locality from which the colony was obtained, while the *y* axis shows the 1C values in picograms (pg). Each letter from (a) to (b) represents a significant group of mean values (p < 0.05) recovered from the contrast analysis between colonies.

The size of the haploid genome of *Mycetophylax conformis* ranged from 0.27 pg to 0.35 pg (Fig 1). The colonies from the Brazilian states of Bahia and Rio de Janeiro were compared statistically, although no significant differences were found between colonies from the same locality (p > 0.05). However, the mean genome sizes were significantly different (p < 0.01) between these two states (Fig 2). The mean genome of the Bahia populations was approximately 0.28 pg (Table 1), with individual values ranging from 0.27 pg to 0.29 pg, whereas the mean of the populations from Rio de Janeiro was approximately 0.33 pg (Table 1), with individual values of between 0.32 pg and 0.35 pg. In *Mycetophylax morschi*, the haploid genomes ranged from 0.32 pg to 0.37 pg (Fig 1).

The colonies from the Brazilian states of Bahia, Rio de Janeiro, Santa Catarina (Araranguá), and Rio Grande do Sul (Torres) were compared using an ANOVA, which found significant variation (p < 0.01) among the states, although no significant variation was found between colonies from the same locality (p > 0.05). The contrast analysis (Table 1, Fig 2) grouped the populations from Bahia (mean = 0.35 pg) and Rio de Janeiro (mean = 0.37 pg) together, as well as the populations from Araranguá, in Santa Catarina (mean = 0.33 pg) and Torres, in Rio Grande do Sul (mean = 0.33 pg).

**Fig 3:**
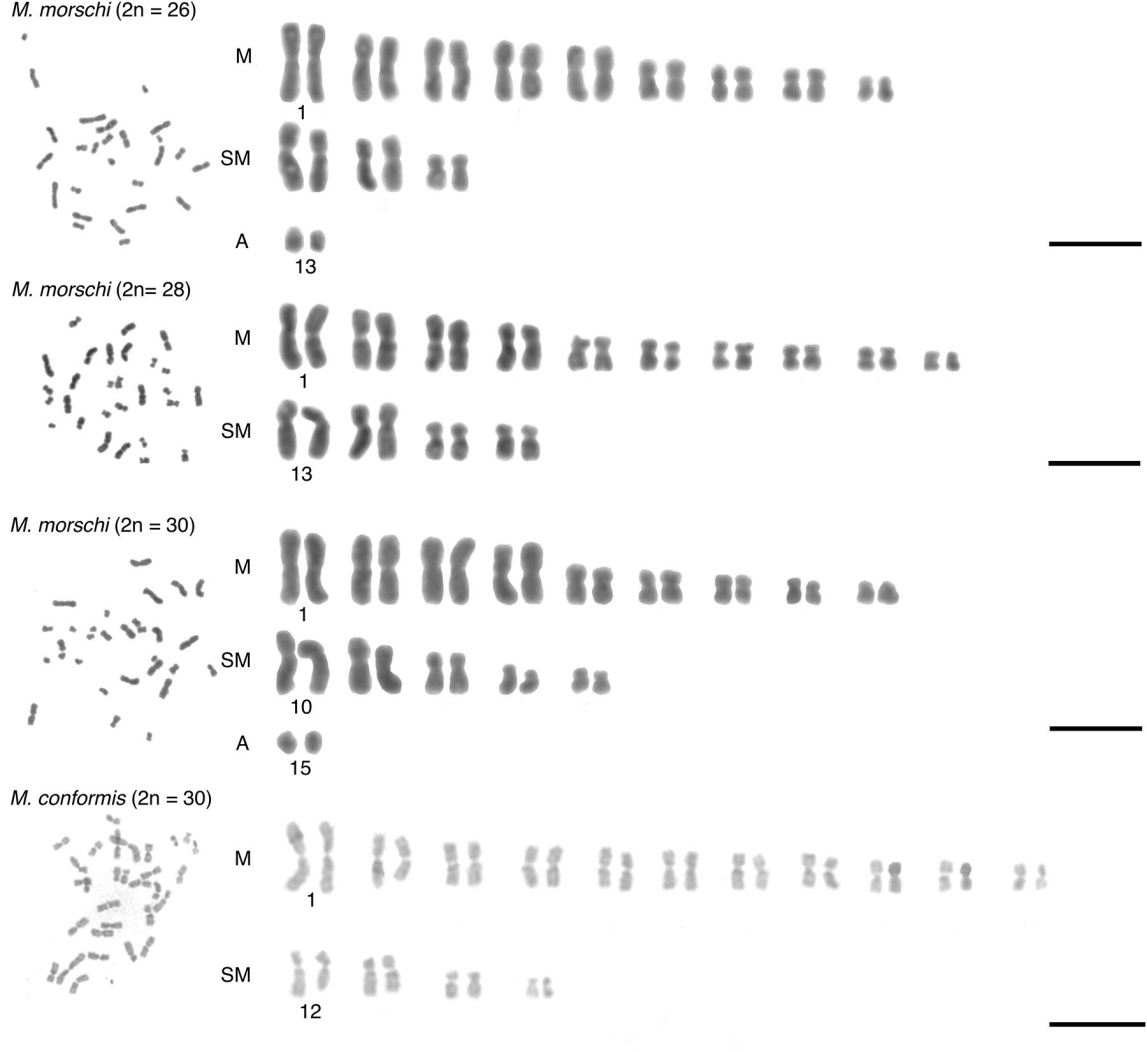
Karyotypes of the *Mycetophylax conformis* and *M*. *morschi* cytotypes, as determined by the karyomorphometric analyses.

The diploid numbers of all *M*. *conformis* individuals analyzed here were 2n = 30, whereas the *M*. *morschi* cytotypes ranged from 2n = 26 in the southern populations (RS and SC), to 2n = 28 in the Bahia population (BA), and 2n = 30 in the Rio de Janeiro (RJ) population (Fig 3). These findings are consistent with those of previous studies (see [11, 17, 47]). No numerical or morphological variation was found among the *M*. *conformis* populations, whereas the number of chromosomes and karyotype did vary among the *M*. *morschi* population, as expected. The karyotype length (the sum of each mean chromosome length in a given karyotype) varied significantly among the populations, and proportionally to the genome size estimated here (Table 3, S1–S4 Tables).

**Table 3:** Karyomorphometric parameters of the *Mycetophylax conformis* and *M*.*morschi* populations analyzed in the present study, showing the diploid number, Karyotype Length (KL), and mean genome size (pg) for each study population.

Species and population	Diploid number	Karyotype length (KL)	Mean GS (pg) ± SD
*Mycetophylax conformis*–BA	2n = 30	90.18 μm	0.28 ± 0.005
*Mycetophylax conformis*–RJ	2n = 30	98.16 μm	0.33 ± 0.013
*Mycetophylax morschi*–RS/SC	2n = 26	72.71 μm	0.33 ± 0.009
*Mycetophylax morschi*–BA	2n = 28	75.83 μm*	0.35 ± 0.008
*Mycetophylax morschi*–RJ	2n = 30	79.80 μm	0.37 ± 0.013

* From Cardoso et al. [47].

#### Internal standards, lysis buffers, and tissue

**Fig 4:**
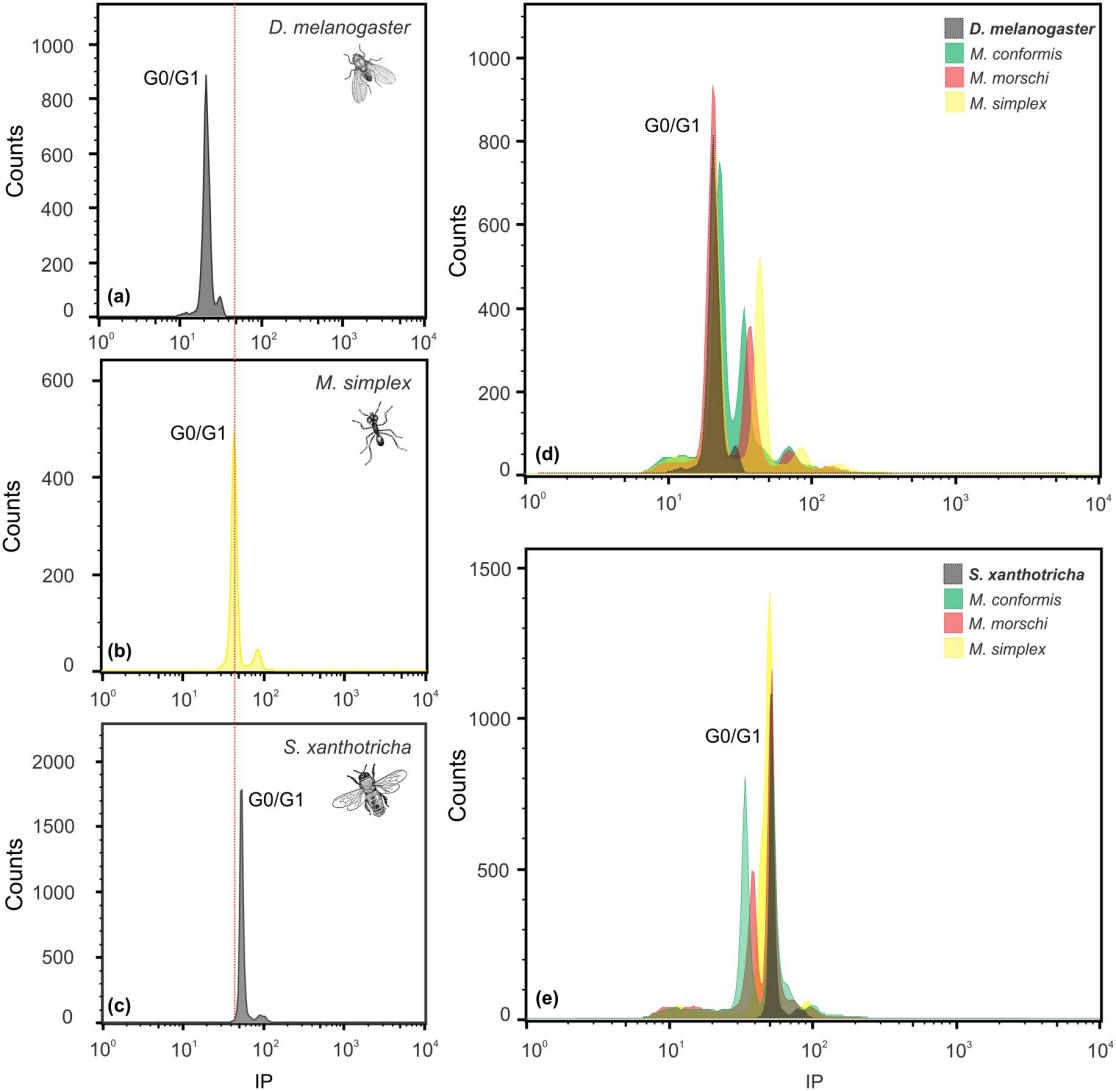
The genome sizes of the *Mycetophylax* species and the internal standards based on the DNA histograms stained with propidium iodide. The nuclei suspensions were obtained with the Galbraith buffer. (a) *M*. *simplex*; (b) *Drosophila melanogaster*; (c) *Scaptotrigona xanthotricha* female, and *M*. *morschi*, *M*. *conformis* and *M*. *simplex* tested with (d) *D*. *melanogaster* and (e) female *S*. *xanthotricha* female as the internal standard. The *x* axis is an arbitrary scale of fluorescence intensity (which is proportional to the size of the genome), and the *y* axis represents the number of nuclei with the specified intensity of fluorescence.

The appropriate internal standard for the FCM analysis depends on the sample. Here we evaluated the two internal standards used widely to estimate genome size in ants and stingless bees. Our analyses indicated that *Drosophila melanogaster* is a better internal standard for ants in comparison with the female *Scaptotrigona xanthotricha* (Fig 4). The peak of the histogram of the estimates of the *S*. *xanthotricha* DNA overlaps with those of the majority of the ants analyzed here (see Fig 1e). However, *D*. *melanogaster* (which 1C = 0.18 pg) has a smaller genome, so its peak does not overlap with those of the other species (Fig 4). The histograms used to infer the genome size (GS) of each specimen presented peaks corresponding to the G_0_/G_1_ and G_2_ phase nuclei of the target species and the internal standard. All the histograms used to calculate the genome size had a high resolution, and their coefficients of variation were always less than 5%, which is considered acceptable for the estimation of GS using FCM [48].

Three buffers commonly used in FCM analyses were tested on all the ants shown in Table 1. The Galbraith buffer was clearly the most effective, based on the CVs of the histogram of the analysis of the species of the subfamilies Myrmicinae and Pseudomyrmecinae. This buffer provided a good resolution, with coefficients of variation invariably lower than 5%, as can be seen in the histograms of the *Mycetophylax* species (Figs 1 and 4). The OTTO buffer (Otto I/Otto II) also worked well, but in the present study, some coefficients of variation were unacceptably large, i.e., greater than 5%. Given this, the data obtained using the OTTO buffer were discarded. The LB01 buffer also worked well for the two ant subfamilies, although the Galbraith buffer was used preferentially in the experiments. Even so, there was no significant difference (p > 0.05) in the mean genome sizes obtained using the Galbraith and LB01 buffers. However, neither the Galbraith nor the OTTO buffers were adequate for the analyses of the species of the subfamilies Ponerinae and Ectatomminae, recovering poor histograms that could not be used to estimate the size of the genome reliably. In these cases, only the LB01 buffer was adequate, providing an excellent resolution with a coefficient of variation of less than 5%.

In the present study, no significant difference was found in the size of the genomes of the adult workers and pupae of the same ant species (p > 0.05). Given this, the estimates obtained for the pupae were included in the calculations of mean genome size shown in Table 1. The “statistical” CV for the genome size of each colony on different measurement days tested the efficacy of the FCM measurements, which were repeated on different days, and the quality control. No significant differences were found between days (p > 0.05), however, which confirmed the precision and reliability of the cytometer. The CVs for each measurement day are plotted in S2 Fig.

## Discussion

### Karyotype and genome size

Chromosomes are the units of inheritance that contain the complete set of information necessary for the development of the organism, and represent the structural organization of the genome. The size of the genome, in turn, provides a quantitative measure of the genome that ultimately expresses the DNA amount of a specimen. Here, we demonstrated once again that differences in karyotype length can represent the variation in the estimates of genome size in either the cytotypes or among populations. In a study of geographically distant populations of the fungus-farming ant *Mycetomoellerius holmgreni* Cardoso et al. [16] suggested that the correlation between genome size and karyotype length is probably due to changes in the centromeric satellite DNA. They also hypothesized that the changes in the karyotypes of the study populations are molded by a process of centromere drive, which favors the evolution of the karyotype and acts as a potential barrier to gene flow. Studies of parasitic wasps of the families Figitidae and Aphelinidae also reported that the variation in the size of the genomes of the study species was correlated with that in the length of the karyotype [26, 27]. In both cases, the authors suggest that the variation in genome size is the result of differences in the relative amount of repetitive DNA sequences, and that only major differences were apparent, probably due to the variation in genome condensation.

A number of studies have shown that genome size varies considerably among species and even between closely-related taxa [49], although only a few studies have focused on, and quantified, the extent of the variation in genome size among populations [16, 50]. Some ecological and physiological traits have been correlated with the size of the genome, *e*.*g*., metabolic rate, genome expression, and cell size [3], and all these traits have a direct effect on the fitness of the organism, and are thus subject to natural selection [51]. However, it is still unclear which factors are the drivers of genome enlargement or shrinkage, although the size of the genome is known to change as a consequence of gene amplification or expression, duplication, and deletion, as well as expansion or contraction of satellite DNA and transposable elements [52]. All these modifications are thought to be triggered by stressful conditions experienced during the dispersal and establishment of species in new habitats [53, 54].

The findings of the present study are consistent with the hypothesis that genome size varies between more ancient lineages and populations founded more recently [53]. Micolino et al. [17] inferred the ancestral area of occurrence and the putative biogeographical dispersal routes of *Mycethopylax* from the southern grasslands of Brazil to the Atlantic coast. In this context, the *Mycetophylax conformis* populations from Bahia (northeastern Brazil) had smaller mean genome sizes than the populations from Rio de Janeiro, southeastern Brazil, apparently established more recently. By contrast, the genome size estimates for *Mycetophylax morschi* from Rio de Janeiro and Bahia were larger than those from the southern Brazilian populations (Santa Catarina and Rio Grande do Sul), and formed a sister clade with these southern populations (see Micolino et al. [17]), which had smaller genomes and karyotype (2n = 26). Similar variation in chromosome length and number among populations has been observed in two social wasps of the genus *Synoeca* within similar area of occurrence on Brazilian Atlantic coast [55]. This pattern thus appears to be recurrent in the Hymenoptera and deserves further investigation.

### Internal standards, lysis buffers, and tissue

Most of the estimates of genome size available for formicides have been obtained using *Drosophila melanogaster* as the internal standard [6, 34], although Cardoso et al. [14] also used females of *Scaptotrigona xantotricha* to analyze hymenopterans, in particular, stingless bees [35–37]. In the present study, *D*. *melanogaster* proved to be a good internal standard for the ants analyzed, and was more appropriate than *S*. *xantotricha*, due primarily to the small 1C value of *D*. *melanogaster*, which avoids potential overlap between the peaks of the target species and the internal standard. When this overlap occurs, it hampers the accurate estimation of the C value [56]. The genome size of *D*. *melanogaster* was first determined by Feulgen densitometry, with 1C being estimated to be 0.18 pg [57, 58]. Given the importance of *D*. *melanogaster* and its ample use in studies of genetics, cytology, and developmental biology [59], a number of subsequent studies were conducted, using different techniques, such as FCM and whole genome sequencing, although they only confirmed the genome size estimates for this organism [60–63].

By contrast, only Lopes et al. [37] and Tavares et al. [35, 36] have estimated the genome size of *Scapitotrigona xantotricha*, and found a difference in the values recorded for males and females. This is probably because bees, like ants, have a haplodiploid sex determination system, with haploid males and diploid females (workers and queens). As ant workers are diploid, it is necessary to use only the female bees to avoid problems of linearity in the measurements (see Lopes et al. [37]). Given this, it is essential to sex the individuals (pupae or adults) prior to the analysis to avoid introducing potential bias. Yet, considering *D*. *melanogaster*, keeping and monitoring the *Drosophila* strain under laboratory conditions should be sufficient to avoid this potential bias. The stability of the *D*. *melanogaster* genome and the relative convenience of using this organism, as shown by the studies mentioned above, reinforce the advantages of using this species as the internal standard for genome size studies in ants. This dipteran also satisfies the guidelines recommended for the FCM analysis [38].

This study is the first to test the different lysis buffers used to obtain nuclei suspensions in ants, which are used to measure the size of the genome of these insects. This type of comparative study is common in plants, and this research has shown that there is no optimum buffer, but rather, that it is necessary to consider the specific characteristics of each study organism when selecting the buffer, to ensure that histograms generated have the lowest possible coefficient of variation, with the least possible background noise [38, 39, 56]. Two of the three buffers tested in the present study were clearly more efficient at extracting nuclei from the ants. These were Galbraith buffer, for the Myrmicinae and Pseudomyrmecinae, and the LB01 buffer, for the Ponerinae and Ectatomminae. The Galbraith buffer has previously been used for ants by Tsutsui et al. [34] and Ardila-Garcia et al. [6], while the OTTO buffers were used by Cardoso et al. [14]. None of these studies tested alternative buffers, however, and did not discuss their choice of buffers, exactly because of a lack of any previous testing in ants. Nevertheless, the genome size values obtained by Cardoso et al. [14] for *Mycetophylax* species using pupae and the OTTO buffer were the same as those obtained in the present study, using the Galbraith buffer and adult workers collected from colonies from the same study locality.

These findings corroborate Loureiro et al. [39], who demonstrated that each buffer contains reagents with different properties that need to be taken into account when selecting a buffer for the analysis of a specific study organism. The LB01 buffer includes mercaptoethanol, which suppresses the negative effects of phenols and other cytosolic compounds during the isolation of the nuclei [38]. We noted that the LB01 buffer, which is widely used in plant studies, achieved good results for the analysis of the ponerine and ectatommine ants. The variation in the cytosol concentrations found in different ant species, and compounds in the exoskeleton and glands in the head may influence the isolation and stability of the nuclei. This means that the careful selection of the buffer is fundamental to ensure obtaining intact nuclei from the worker ant cells used in the FCM analysis, thus preventing DNA degradation and guaranteeing stoichiometric staining [38].

The present study also compared the sizes of the genomes of adult worker ants and the pupae for the first time, and found no significant differences between them. This is a very important advance in the FCM analysis of ants, given that collecting adult individuals is less labor-intensive than extracting the entire colony. For previous studies, it was usually necessary to obtain the brood, which depends on the life cycle of the colony and the time of the year (see Cardoso et al. [14]). Further, Doležel and Bartoš [38] demonstrated that one of the difficulties in the use of FCM to analyze plants was the need to obtain fresh or live tissue, and this was confirmed here for the FCM analysis of ants. We would recommend obtaining nuclei for FCM analysis from freshly-sampled or living material, due the superior quality of the isolated nuclei, in order to guarantee satisfactory results.

## Conclusions

The results of the present study on the variation in karyotype length and genome size among *M*. *morschi* and *M*. *conformis* populations revealed hidden diversity that has mostly been overlooked in traditional descriptions of karyotypes. The changes observed in the fine structure of the chromosomes may represent the first steps in the process of karyotype evolution, as suggested for *Mycetomoellerius holmgreni* [16]. The genome sizes estimated in the present study varied among cytotypes and populations. This variation is likely to be related primarily to the stress experience during the dispersal of the species to new areas. We also provide detailed methodological recommendations for the standardization of the procedure used to quantify the size of the genome in ants by using the head of adult workers rather than tissue from the larvae or pupae, and discuss the ideal internal standard reference. We also found that different lysis buffers are more appropriate for the analysis of different ant subfamilies. It is thus necessary to select the optimal lysis buffer to ensure the adequate suspension of nuclei when using adult workers as the source of tissue. The FCM also proved, once more, to be an effective method for the quantification of DNA, with no significant variation being found among measurement days.

## Supporting information

S1 Fig Genome size DNA-histograms of *Scaptotrigona xantotricha* and *Drosophila melanogaster* (Oregon-R) reared at Universidade Federal de Ouro Preto through analysis of nuclear suspensions of cerebral ganglion tissue, stained with PI. Female of *S*. *xantotricha* (used as internal standard 2C = 0.88 pg, channel 200) and *D*. *melanogaster* (2C = 0.36 pg, channel 100).
(TIF)

S2 Fig Coefficient of variation (CV) of genome size calculated per day of measurement on the flow cytometer. Calculate using the sampled colonies of the four Myrmicinae species with *D*. *melanogaster* as internal standard and Galbraith’s buffer.
(TIF)

S1 Table Karyomorphometric analyses of the chromosomes of *Mycetophylax conformis* (Rio de Janeiro) 2n = 30. (DOCX)

S2 Table Karyomorphometric analyses of the chromosomes of *Mycetophylax morschi* (Santa Catarina) 2n = 26. (DOCX)

S3 Table Karyomorphometric analyses of the chromosomes of *Mycetophylax morschi* (Bahia) 2n = 28. (DOCX)

S4 Table Karyomorphometric analyses of the chromosomes of *Mycetophylax morschi* (Rio de Janeiro) 2n = 30. (DOCX)
