# Supplementary figures and images for: Intraspecific variation in the karyotype length and genome size of fungus-farming ants (genus Mycetophylax), with remarks on procedures for the estimation of genome size in the Formicidae by flow cytometry
Source: PLoS One. 2020 Aug 6;15(8):e0237157. doi: 10.1371/journal.pone.0237157 (PMC7410318; doi:10.1371/journal.pone.0237157)

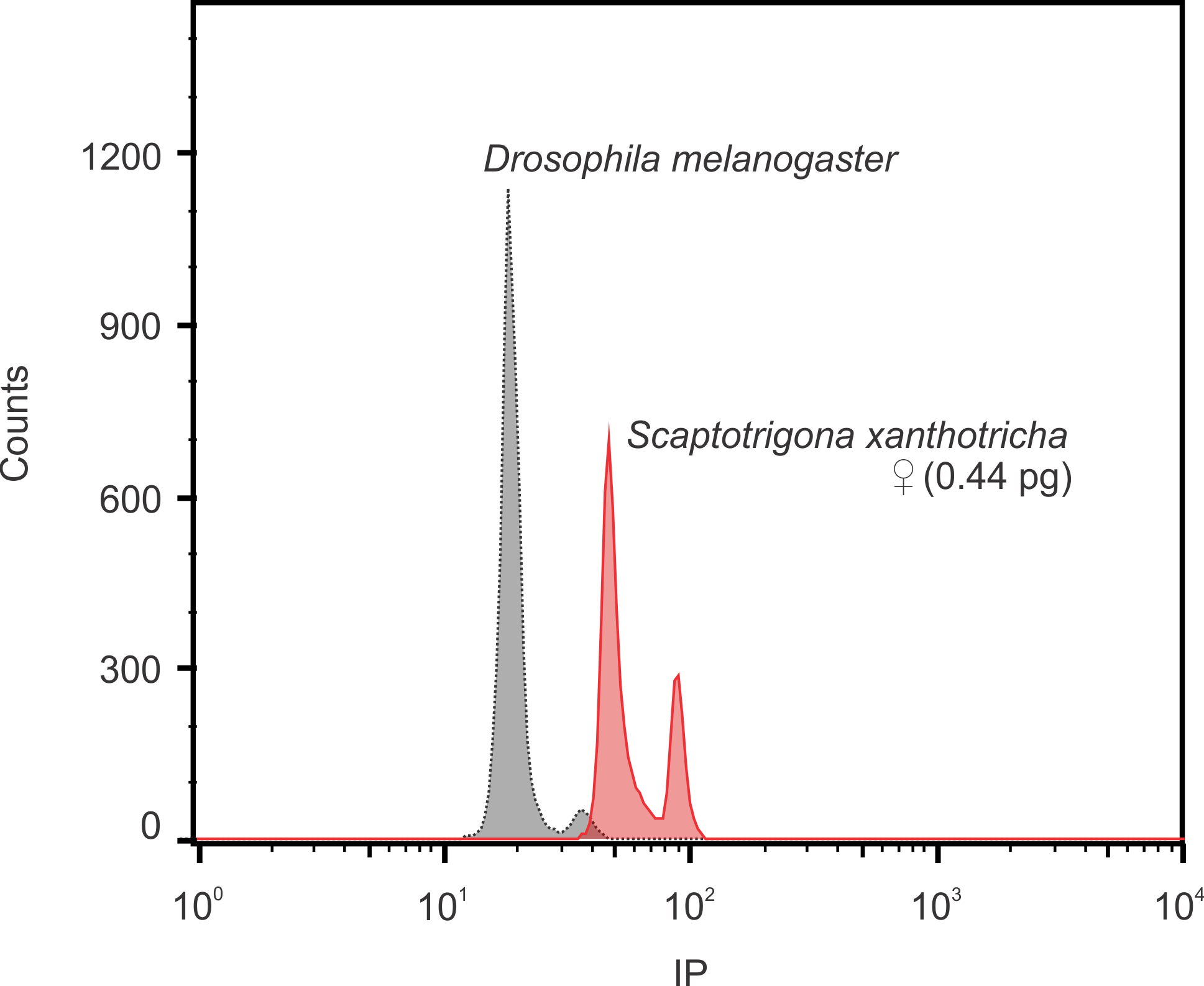

Supplement: S1 Fig — Female of S. xantotricha (used as internal standard 2C = 0.88 pg, channel 200) and D. melanogaster (2C = 0.36 pg, channel 100). (TIF) [file pone.0237157.s001.tif]

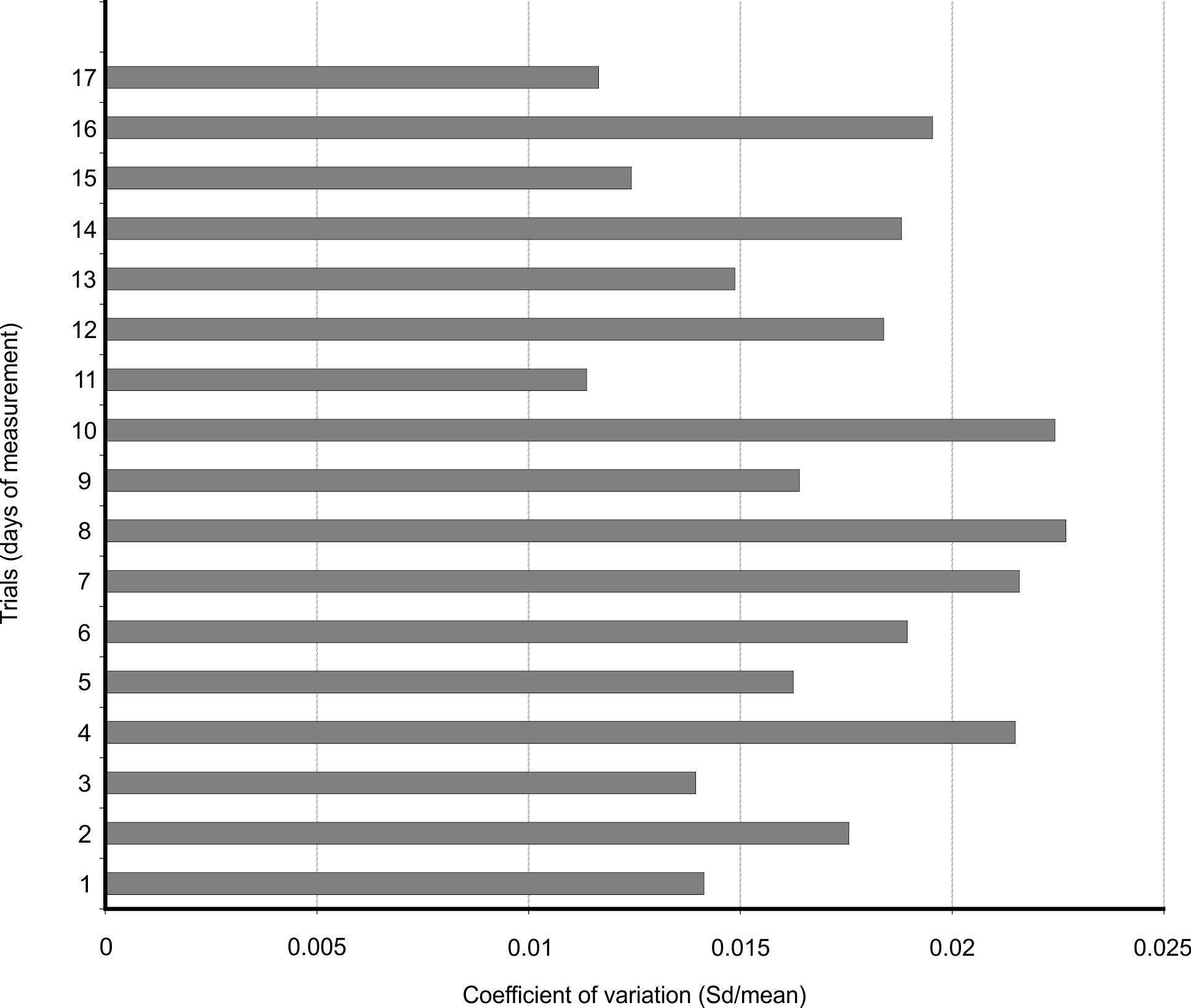

Supplement: S2 Fig — Calculate using the sampled colonies of the four Myrmicinae species with D. melanogaster as internal standard and Galbraith’s buffer. (TIF) [file pone.0237157.s002.tif]
