# Supplementary material for: Intraspecific variation in the karyotype length and genome size of fungus-farming ants (genus Mycetophylax), with remarks on procedures for the estimation of genome size in the Formicidae by flow cytometry
Source: PLoS One. 2020 Aug 6;15(8):e0237157. doi: 10.1371/journal.pone.0237157 (PMC7410318; doi:10.1371/journal.pone.0237157)
Supplement: S1 Table — (DOCX) [file pone.0237157.s003.docx]

**S1 Table.** Karyomorphometric analyses of the chromosomes of *Mycetophylax* *conformis* (Rio de Janeiro) 2n=30.

| **Chromosome** | **TL(µM)** | **L(µM)** | **S(µM)** | **RL(µM)** | ***r*** | **Classification** |
| --- | --- | --- | --- | --- | --- | --- |
| 1 | 6.49±0.21 | 3.47±0.35 | 2.99±0.09 | 6.61±0.01 | 1.16±0.15 | Metacêntrico |
| 1 | 6.34±0.05 | 3.55±0.23 | 2.80±0.18 | 6.46±0.15 | 1.27±0.16 | Metacêntrico |
| 2 | 4.55±0.36 | 2.41±0.32 | 2.14±0.04 | 4.64±0.51 | 1.12±0.13 | Metacêntrico |
| 2 | 4.12±0.03 | 2.22±0.08 | 1.91±0.05 | 4.20±0.10 | 1.16±0.07 | Metacêntrico |
| 3 | 3.89±0.21 | 2.03±0.18 | 1.86±0.02 | 3.96±0.09 | 1.09±0.08 | Metacêntrico |
| 3 | 3.83±0.24 | 2.10±0.09 | 1.73±0.15 | 3.90±0.12 | 1.21±0.05 | Metacêntrico |
| 4 | 3.55±0.04 | 1.99±0.00 | 1.56±0.04 | 3.62±0.16 | 1.28±0.03 | Metacêntrico |
| 4 | 3.53±0.02 | 1.83±0.01 | 1.70±0.02 | 3.60±0.13 | 1.07±0.02 | Metacêntrico |
| 5 | 3.45±0.09 | 1.89±0.09 | 1.53±0.05 | 3.52±0.02 | 1.24±0.11 | Metacêntrico |
| 5 | 3.34±0.17 | 1.86±0.04 | 1.48±0.13 | 3.40±0.07 | 1.26±0.08 | Metacêntrico |
| 6 | 3.30±0.15 | 1.79±0.05 | 1.51±0.20 | 3.36±0.05 | 1.20±0.19 | Metacêntrico |
| 6 | 3.26±0.09 | 1.86±0.05 | 1.40±0.04 | 3.32±0.01 | 1.33±0.01 | Metacêntrico |
| 7 | 3.08±0.13 | 1.85±0.03 | 1.26±0.05 | 3.14±0.03 | 1.47±0.04 | Metacêntrico |
| 7 | 3.00±0.24 | 1.67±0.15 | 1.33±0.09 | 3.05±0.15 | 1.25±0.04 | Metacêntrico |
| 8 | 2.92±0.21 | 1.60±0.07 | 1.29±0.19 | 2.97±0.12 | 1.26±0.14 | Metacêntrico |
| 8 | 2.75±0.06 | 1.60±0.06 | 1.16±0.12 | 2.81±0.02 | 1.38±0.19 | Metacêntrico |
| 9 | 2.71±0.01 | 1.50±0.01 | 1.22±0.00 | 2.76±0.08 | 1.23±0.01 | Metacêntrico |
| 9 | 2.65±0.00 | 1.49±0.10 | 1.16±0.10 | 2.70±0.08 | 1.30±0.19 | Metacêntrico |
| 10 | 2.53±0.10 | 1.40±0.15 | 1.13±0.05 | 2.57±0.02 | 1.24±0.18 | Metacêntrico |
| 10 | 2.26±0.02 | 1.11±0.12 | 1.15±0.09 | 2.31±0.05 | 1.14±0.05 | Metacêntrico |
| 11 | 2.24±0.00 | 1.25±0.15 | 0.99±0.14 | 2.29±0.07 | 1.29±0.33 | Metacêntrico |
| 11 | 2.07±0.04 | 1.11±0.04 | 0.96±0.08 | 2.11±0.11 | 1.17±0.14 | Metacêntrico |
| 12 | 4.03±0.26 | 2.67±0.14 | 1.37±0.11 | 4.11±0.13 | 1.96±0.06 | Submetacêntrico |
| 12 | 3.65±0.45 | 2.47±0.40 | 1.18±0.05 | 3.71±0.34 | 2.09±0.24 | Submetacêntrico |
| 13 | 3.36±0.31 | 2.27±0.14 | 1.12±0.12 | 3.42±0.21 | 2.03±0.10 | Submetacêntrico |
| 13 | 3.03±0.12 | 1.97±0.01 | 1.03±0.06 | 3.09±0.21 | 1.93±0.11 | Submetacêntrico |
| 14 | 2.55±0.25 | 1.73±0.26 | 0.81±0.01 | 2.59±0.17 | 2.13±0.34 | Submetacêntrico |
| 14 | 2.31±0.21 | 1.65±0.12 | 0.67±0.08 | 2.35±0.14 | 2.46±0.13 | Submetacêntrico |
| 15 | 1.71±0.10 | 1.16±0.00 | 0.55±0.10 | 1.74±0.05 | 2.14±0.40 | Submetacêntrico |
| 15 | 1.66±0.05 | 1.15±0.00 | 0.51±0.05 | 1.69±0.00 | 2.26±0.21 | Submetacêntrico |
| **∑** | 98.16 |  |  |  |  |  |

**TL**: total length; **L**: long arm length; **S**: short arm length; **RL**: relative length; **r**: arm ratio (= L/S).
