# Supplementary material for: Intraspecific variation in the karyotype length and genome size of fungus-farming ants (genus Mycetophylax), with remarks on procedures for the estimation of genome size in the Formicidae by flow cytometry
Source: PLoS One. 2020 Aug 6;15(8):e0237157. doi: 10.1371/journal.pone.0237157 (PMC7410318; doi:10.1371/journal.pone.0237157)
Supplement: S2 Table — (DOCX) [file pone.0237157.s004.docx]

**S2 Table.** Karyomorphometric analyses of the chromosomes of *Mycetophylax* *morschi* (Santa Catarina) 2n=26.

| **Chromosome** | **TL(µM)** | **L(µM)** | **S(µM)** | **RL(µM)** | ***r*** | **Classification** |
| --- | --- | --- | --- | --- | --- | --- |
| 1 | 4.53±0.48 | 2.41±0.24 | 2.12±0.25 | 6.20±0.30 | 1.15±0.09 | Metacêntrico |
| 1 | 4.29±0.51 | 2.31±0.26 | 1.98±0.27 | 5.88±0.18 | 1.16±0.06 | Metacêntrico |
| 2 | 3.95±0.43 | 2.34±0.31 | 1.61±0.16 | 5.45±0.19 | 1.44±0.17 | Metacêntrico |
| 2 | 3.61±0.46 | 2.04±0.33 | 1.56±0.20 | 4.94±0.19 | 1.39±0.17 | Metacêntrico |
| 3 | 3.48±0.38 | 1.93±0.20 | 1.55±0.21 | 4.77±0.11 | 1.27±0.19 | Metacêntrico |
| 3 | 3.35±0.40 | 1.87±0.23 | 1.48±0.21 | 4.62±0.12 | 1.31±0.12 | Metacêntrico |
| 4 | 3.26±0.34 | 1.84±0.19 | 1.42±0.2 | 4.48±0.13 | 1.30±0.14 | Metacêntrico |
| 4 | 3.15±0.34 | 1.74±0.18 | 1.41±0.22 | 4.32±0.19 | 1.22±0.17 | Metacêntrico |
| 5 | 3.01±0.41 | 1.69±0.24 | 1.32±0.21 | 4.21±0.18 | 1.33±0.20 | Metacêntrico |
| 5 | 2.84±0.34 | 1.59±0.24 | 1.25±0.17 | 4.02±0.33 | 1.32±0.16 | Metacêntrico |
| 6 | 2.31±0.3 | 1.35±0.21 | 0.96±0.11 | 3.22±0.16 | 1.38±0.15 | Metacêntrico |
| 6 | 2.23±0.28 | 1.28±0.19 | 0.95±0.12 | 3.11±0.19 | 1.37±0.17 | Metacêntrico |
| 7 | 2.11±0.22 | 1.15±0.15 | 0.95±0.11 | 2.94±0.13 | 1.25±0.18 | Metacêntrico |
| 7 | 2.02±0.21 | 1.14±0.15 | 0.88±0.10 | 2.79±0.09 | 1.32±0.21 | Metacêntrico |
| 8 | 1.96±0.18 | 1.11±0.11 | 0.85±0.08 | 2.71±0.10 | 1.34±0.18 | Metacêntrico |
| 8 | 1.82±0.23 | 1.03±0.12 | 0.79±0.13 | 2.56±0.09 | 1.31±0.13 | Metacêntrico |
| 9 | 1.52±0.18 | 0.92±0.11 | 0.59±0.08 | 2.12±0.19 | 1.53±0.10 | Metacêntrico |
| 9 | 1.40±0.17 | 0.84±0.10 | 0.56±0.08 | 1.93±0.11 | 1.52±0.07 | Metacêntrico |
| 10 | 3.91±0.32 | 2.48±0.19 | 1.43±0.14 | 5.36±0.22 | 1.72±0.05 | Submetacêntrico |
| 10 | 3.70±0.34 | 2.36±0.20 | 1.34±0.15 | 5.05±0.17 | 1.87±0.17 | Submetacêntrico |
| 11 | 3.47±0.38 | 2.23±0.23 | 1.24±0.16 | 4.72±0.27 | 1.80±0.15 | Submetacêntrico |
| 11 | 3.32±0.40 | 2.12±0.27 | 1.20±0.14 | 4.51±0.32 | 1.71±0.03 | Submetacêntrico |
| 12 | 2.46±0.50 | 1.59±0.33 | 0.88±0.17 | 3.26±0.34 | 1.77±0.13 | Submetacêntrico |
| 12 | 2.20±0.20 | 1.44±0.18 | 0.76±0.06 | 3.00±0.10 | 1.92±0.39 | Submetacêntrico |
| 13 | 1.47±0.16 | 1.30±0.14 | 0.17±0.02 | 2.02±0.10 | 8.03±0.63 | Acrocêntrico |
| 13 | 1.34±0.15 | 1.18±0.13 | 0.16±0.02 | 1.83±0.12 | 7.73±0.47 | Acrocêntrico |
| **∑** | 72.71 |  |  |  |  |  |

**TL**: total length; **L**: long arm length; **S**: short arm length; **RL**: relative length; **r**: arm ratio (= L/S).
