# Supplementary material for: Intraspecific variation in the karyotype length and genome size of fungus-farming ants (genus Mycetophylax), with remarks on procedures for the estimation of genome size in the Formicidae by flow cytometry
Source: PLoS One. 2020 Aug 6;15(8):e0237157. doi: 10.1371/journal.pone.0237157 (PMC7410318; doi:10.1371/journal.pone.0237157)
Supplement: S3 Table — (DOCX) [file pone.0237157.s005.docx]

**S3 Table**. Karyomorphometric analyses of the chromosomes of *Mycetophylax* *morschi* (Bahia) 2n=28.

| **Chromosome** | **TL(µM)** | **L(µM)** | **S(µM)** | **RL(µM)** | ***r*** | **Classification** |
| --- | --- | --- | --- | --- | --- | --- |
| 1 | 4.41±1.40 | 2.33±0.69 | 2.09±0.73 | 5.75±0.42 | 1.14±0.19 | Metacêntrico |
| 1 | 4.28±1.32 | 2.28±0.62 | 2.00±0.72 | 5.58±0.39 | 1.20±0.23 | Metacêntrico |
| 2 | 3.84±0.97 | 2.16±0.53 | 1.69±0.46 | 5.05±0.08 | 1.18±0.13 | Metacêntrico |
| 2 | 3.71±0.88 | 2.11±0.57 | 1.60±0.33 | 4.89±0.07 | 1.32±0.19 | Metacêntrico |
| 3 | 3.69±0.99 | 2.02±0.55 | 1.67±0.47 | 4.84±0.12 | 1.19±0.14 | Metacêntrico |
| 3 | 3.51±0.98 | 1.93±0.56 | 1.58±0.42 | 4.60±0.20 | 1.22±0.18 | Metacêntrico |
| 4 | 3.28±0.98 | 1.92±0.56 | 1.36±0.43 | 4.28±0.36 | 1.40±0.21 | Metacêntrico |
| 4 | 3.08±0.94 | 1.85±0.56 | 1.23±0.40 | 4.02±0.33 | 1.47±0.10 | Metacêntrico |
| 5 | 2.57±0.93 | 1.55±0.57 | 1.03±0.36 | 3.33±0.34 | 1.46±0.18 | Metacêntrico |
| 5 | 2.29±0.64 | 1.30±0.28 | 0.98±0.36 | 3.00±0.14 | 1.36±0.20 | Metacêntrico |
| 6 | 2.17±0.62 | 1.24±0.37 | 0.93±0.27 | 2.84±0.12 | 1.32±0.19 | Metacêntrico |
| 6 | 2.11±0.64 | 1.16±0.35 | 0.95±0.29 | 2.76±0.17 | 1.40±0.17 | Metacêntrico |
| 7 | 2.00±0.42 | 1.12±0.22 | 0.88±0.21 | 2.65±0.13 | 1.32±0.14 | Metacêntrico |
| 7 | 1.78±0.31 | 0.98±0.21 | 0.80±0.13 | 2.37±0.17 | 1.39±0.19 | Metacêntrico |
| 8 | 1.71±0.22 | 0.94±0.20 | 0.77±0.04 | 2.30±0.22 | 1.32±0.25 | Metacêntrico |
| 8 | 1.63±0.26 | 0.92±0.12 | 0.71±0.15 | 2.18±0.21 | 1.45±0.20 | Metacêntrico |
| 9 | 1.59±0.27 | 0.90±0.15 | 0.69±0.13 | 2.12±0.16 | 1.34±0.23 | Metacêntrico |
| 9 | 1.51±0.16 | 0.86±0.11 | 0.65±0.06 | 2.04±0.26 | 1.26±0.17 | Metacêntrico |
| 10 | 1.38±0.23 | 0.81±0.18 | 0.57±0.09 | 1.85±0.26 | 1.50±0.08 | Metacêntrico |
| 10 | 1.28±0.15 | 0.75±0.07 | 0.53±0.09 | 1.73±0.21 | 1.42±0.12 | Metacêntrico |
| 11 | 3.90±1.08 | 2.53±0.71 | 1.37±0.38 | 5.11±0.21 | 1.85±0.11 | Submetacêntrico |
| 11 | 3.74±1.00 | 2.47±0.68 | 1.27±0.33 | 4.91±0.21 | 1.89±0.25 | Submetacêntrico |
| 12 | 3.66±0.99 | 2.37±0.63 | 1.29±0.37 | 4.8±0.15 | 1.77±0.13 | Submetacêntrico |
| 12 | 3.29±0.41 | 2.14±0.30 | 1.15±0.11 | 4.43±0.52 | 1.82±0.15 | Submetacêntrico |
| 13 | 2.50±0.43 | 1.63±0.31 | 0.87±0.12 | 3.34±0.36 | 1.91±0.29 | Submetacêntrico |
| 13 | 2.40±0.46 | 1.56±0.28 | 0.84±0.18 | 3.19±0.13 | 1.85±0.11 | Submetacêntrico |
| 14 | 2.31±0.37 | 1.53±0.27 | 0.78±0.11 | 3.08±0.21 | 1.97±0.15 | Submetacêntrico |
| 14 | 2.21±0.40 | 1.43±0.23 | 0.78±0.17 | 2.95±0.24 | 2.09±0.32 | Submetacêntrico |
| **∑** | 75.83 |  |  |  |  |  |

**TL**: total length; **L**: long arm length; **S**: short arm length; **RL**: relative length; **r**: arm ratio (= L/S).

**Table S4** - Karyomorphometric analyses of the chromosomes of *Mycetophylax* *morschi* (Rio de Janeiro) 2n=30.

| **Chromosome** | **TL(µM)** | **L(µM)** | **S(µM)** | **RL(µM)** | ***r*** | **Classification** |
| --- | --- | --- | --- | --- | --- | --- |
| 1 | 4.55±0.39 | 2.39±0.22 | 2.15±0.19 | 5.69±0.20 | 1.09±0.07 | Metacêntrico |
| 1 | 4.36±0.38 | 2.32±0.29 | 2.05±0.13 | 5.46±0.20 | 1.13±0.06 | Metacêntrico |
| 2 | 4.24±0.38 | 2.28±0.17 | 1.96±0.24 | 5.30±0.17 | 1.20±0.13 | Metacêntrico |
| 2 | 4.14±0.39 | 2.27±0.25 | 1.87±0.18 | 5.18±0.18 | 1.24±0.10 | Metacêntrico |
| 3 | 3.95±0.30 | 2.13±0.18 | 1.82±0.13 | 4.94±0.12 | 1.25±0.19 | Metacêntrico |
| 3 | 3.84±0.18 | 2.05±0.11 | 1.79±0.10 | 4.81±0.21 | 1.20±0.13 | Metacêntrico |
| 4 | 3.47±0.60 | 1.87±0.25 | 1.60±0.36 | 4.34±0.65 | 1.21±0.18 | Metacêntrico |
| 4 | 3.19±0.56 | 1.75±0.27 | 1.45±0.31 | 3.99±0.64 | 1.21±0.17 | Metacêntrico |
| 5 | 2.41±0.25 | 1.35±0.14 | 1.06±0.15 | 3.02±0.23 | 1.33±0.13 | Metacêntrico |
| 5 | 2.20±0.19 | 1.29±0.15 | 0.91±0.07 | 2.75±0.13 | 1.36±0.10 | Metacêntrico |
| 6 | 2.10±0.22 | 1.22±0.15 | 0.87±0.13 | 2.63±0.14 | 1.36±0.12 | Metacêntrico |
| 6 | 2.00±0.24 | 1.15±0.10 | 0.85±0.15 | 2.50±0.17 | 1.34±0.19 | Metacêntrico |
| 7 | 1.76±0.11 | 1.00±0.10 | 0.76±0.06 | 2.21±0.07 | 1.31±0.10 | Metacêntrico |
| 7 | 1.72±0.10 | 0.97±0.12 | 0.76±0.05 | 2.16±0.11 | 1.23±0.19 | Metacêntrico |
| 8 | 1.69±0.12 | 0.96±0.07 | 0.74±0.10 | 2.12±0.12 | 1.30±0.17 | Metacêntrico |
| 8 | 1.58±0.13 | 0.89±0.09 | 0.69±0.05 | 1.98±0.09 | 1.36±0.20 | Metacêntrico |
| 9 | 1.49±0.12 | 0.88±0.10 | 0.61±0.07 | 1.87±0.07 | 1.34±0.16 | Metacêntrico |
| 9 | 1.39±0.06 | 0.82±0.05 | 0.57±0.05 | 1.75±0.08 | 1.40±0.19 | Metacêntrico |
| 10 | 3.99±0.36 | 2.54±0.25 | 1.45±0.12 | 5.00±0.25 | 1.80±0.13 | Submetacêntrico |
| 10 | 3.77±0.23 | 2.42±0.16 | 1.35±0.08 | 4.72±0.08 | 1.80±0.15 | Submetacêntrico |
| 11 | 3.48±0.20 | 2.21±0.15 | 1.27±0.07 | 4.37±0.18 | 1.79±0.12 | Submetacêntrico |
| 11 | 3.32±0.25 | 2.12±0.18 | 1.20±0.11 | 4.16±0.24 | 1.76±0.08 | Submetacêntrico |
| 12 | 2.67±0.33 | 1.71±0.20 | 0.96±0.13 | 3.36±0.47 | 1.85±0.11 | Submetacêntrico |
| 12 | 2.58±0.28 | 1.67±0.18 | 0.91±0.11 | 3.25±0.44 | 1.82±0.16 | Submetacêntrico |
| 13 | 2.10±0.21 | 1.35±0.15 | 0.74±0.08 | 2.63±0.32 | 1.83±0.13 | Submetacêntrico |
| 13 | 1.87±0.19 | 1.22±0.12 | 0.65±0.10 | 2.35±0.30 | 1.97±0.17 | Submetacêntrico |
| 14 | 1.65±0.12 | 1.08±0.09 | 0.57±0.05 | 2.07±0.13 | 2.06±0.29 | Submetacêntrico |
| 14 | 1.55±0.17 | 0.99±0.11 | 0.55±0.06 | 1.94±0.18 | 2.03±0.25 | Submetacêntrico |
| 15 | 1.40±0.11 | 1.23±0.10 | 0.17±0.01 | 1.76±0.19 | 8.00±0.60 | Acrocêntrico |
| 15 | 1.34±0.12 | 1.19±0.11 | 0.15±0.01 | 1.69±0.19 | 8.00±0.49 | Acrocêntrico |
| **∑** | 79.8 |  |  |  |  |  |

**TL**: total length; **L**: long arm length; **S**: short arm length; **RL**: relative length; **r**: arm ratio (= L/S).
