# Supplementary material for: Intraspecific variation in the karyotype length and genome size of fungus-farming ants (genus Mycetophylax), with remarks on procedures for the estimation of genome size in the Formicidae by flow cytometry
Source: PLoS One. 2020 Aug 6;15(8):e0237157. doi: 10.1371/journal.pone.0237157 (PMC7410318; doi:10.1371/journal.pone.0237157)
Supplement: S4 Table — (DOCX) [file pone.0237157.s006.docx]

**S4 Table**. Karyomorphometric analyses of the chromosomes of *Mycetophylax* *morschi* (Rio de Janeiro) 2n=30.

| **Chromosome** | **TL(µM)** | **L(µM)** | **S(µM)** | **RL(µM)** | ***r*** | **Classification** |
| --- | --- | --- | --- | --- | --- | --- |
| 1 | 4.55±0.39 | 2.39±0.22 | 2.15±0.19 | 5.69±0.20 | 1.09±0.07 | Metacêntrico |
| 1 | 4.36±0.38 | 2.32±0.29 | 2.05±0.13 | 5.46±0.20 | 1.13±0.06 | Metacêntrico |
| 2 | 4.24±0.38 | 2.28±0.17 | 1.96±0.24 | 5.30±0.17 | 1.20±0.13 | Metacêntrico |
| 2 | 4.14±0.39 | 2.27±0.25 | 1.87±0.18 | 5.18±0.18 | 1.24±0.10 | Metacêntrico |
| 3 | 3.95±0.30 | 2.13±0.18 | 1.82±0.13 | 4.94±0.12 | 1.25±0.19 | Metacêntrico |
| 3 | 3.84±0.18 | 2.05±0.11 | 1.79±0.10 | 4.81±0.21 | 1.20±0.13 | Metacêntrico |
| 4 | 3.47±0.60 | 1.87±0.25 | 1.60±0.36 | 4.34±0.65 | 1.21±0.18 | Metacêntrico |
| 4 | 3.19±0.56 | 1.75±0.27 | 1.45±0.31 | 3.99±0.64 | 1.21±0.17 | Metacêntrico |
| 5 | 2.41±0.25 | 1.35±0.14 | 1.06±0.15 | 3.02±0.23 | 1.33±0.13 | Metacêntrico |
| 5 | 2.20±0.19 | 1.29±0.15 | 0.91±0.07 | 2.75±0.13 | 1.36±0.10 | Metacêntrico |
| 6 | 2.10±0.22 | 1.22±0.15 | 0.87±0.13 | 2.63±0.14 | 1.36±0.12 | Metacêntrico |
| 6 | 2.00±0.24 | 1.15±0.10 | 0.85±0.15 | 2.50±0.17 | 1.34±0.19 | Metacêntrico |
| 7 | 1.76±0.11 | 1.00±0.10 | 0.76±0.06 | 2.21±0.07 | 1.31±0.10 | Metacêntrico |
| 7 | 1.72±0.10 | 0.97±0.12 | 0.76±0.05 | 2.16±0.11 | 1.23±0.19 | Metacêntrico |
| 8 | 1.69±0.12 | 0.96±0.07 | 0.74±0.10 | 2.12±0.12 | 1.30±0.17 | Metacêntrico |
| 8 | 1.58±0.13 | 0.89±0.09 | 0.69±0.05 | 1.98±0.09 | 1.36±0.20 | Metacêntrico |
| 9 | 1.49±0.12 | 0.88±0.10 | 0.61±0.07 | 1.87±0.07 | 1.34±0.16 | Metacêntrico |
| 9 | 1.39±0.06 | 0.82±0.05 | 0.57±0.05 | 1.75±0.08 | 1.40±0.19 | Metacêntrico |
| 10 | 3.99±0.36 | 2.54±0.25 | 1.45±0.12 | 5.00±0.25 | 1.80±0.13 | Submetacêntrico |
| 10 | 3.77±0.23 | 2.42±0.16 | 1.35±0.08 | 4.72±0.08 | 1.80±0.15 | Submetacêntrico |
| 11 | 3.48±0.20 | 2.21±0.15 | 1.27±0.07 | 4.37±0.18 | 1.79±0.12 | Submetacêntrico |
| 11 | 3.32±0.25 | 2.12±0.18 | 1.20±0.11 | 4.16±0.24 | 1.76±0.08 | Submetacêntrico |
| 12 | 2.67±0.33 | 1.71±0.20 | 0.96±0.13 | 3.36±0.47 | 1.85±0.11 | Submetacêntrico |
| 12 | 2.58±0.28 | 1.67±0.18 | 0.91±0.11 | 3.25±0.44 | 1.82±0.16 | Submetacêntrico |
| 13 | 2.10±0.21 | 1.35±0.15 | 0.74±0.08 | 2.63±0.32 | 1.83±0.13 | Submetacêntrico |
| 13 | 1.87±0.19 | 1.22±0.12 | 0.65±0.10 | 2.35±0.30 | 1.97±0.17 | Submetacêntrico |
| 14 | 1.65±0.12 | 1.08±0.09 | 0.57±0.05 | 2.07±0.13 | 2.06±0.29 | Submetacêntrico |
| 14 | 1.55±0.17 | 0.99±0.11 | 0.55±0.06 | 1.94±0.18 | 2.03±0.25 | Submetacêntrico |
| 15 | 1.40±0.11 | 1.23±0.10 | 0.17±0.01 | 1.76±0.19 | 8.00±0.60 | Acrocêntrico |
| 15 | 1.34±0.12 | 1.19±0.11 | 0.15±0.01 | 1.69±0.19 | 8.00±0.49 | Acrocêntrico |
| **∑** | 79.8 |  |  |  |  |  |

**TL**: total length; **L**: long arm length; **S**: short arm length; **RL**: relative length; **r**: arm ratio (= L/S).
